# A novel pathogenic frameshift variant in *AXIN2* in a man with polyposis and hypodontia

**DOI:** 10.1186/s13053-023-00260-6

**Published:** 2023-08-25

**Authors:** M. F. Broekema, E. J. W. Redeker, M. T. Uiterwaal, L. P. van Hest

**Affiliations:** 1 Department of Human Genetics, Amsterdam UMC, Academic Medical Center, Amsterdam, The Netherlands; 2 Department of Gastroenterology, Spaarne Hospital, Hoofddorp, The Netherlands

**Keywords:** AXIN2, WNT signaling, Oligodontia, Hypodontia, Polyposis, Polyps, Colorectal cancer

## Abstract

**Background:**

WNT signaling is pivotal in embryogenesis and tissue homeostasis. Aberrant WNT signaling, due to mutations in components of this pathway, contributes to the development and progression of human cancers, including colorectal cancer. AXIN2, encoded by the *AXIN2* gene, is a key negative regulator and target of the canonical WNT signaling pathway. Germline mutations in *AXIN2* are associated with absence of permanent teeth (hypo- and oligodontia) and predisposition to gastrointestinal polyps and cancer. The limited number of patients makes an accurate genotype–phenotype analysis currently challenging.

**Case presentation:**

We present the case of a 55-year-old male with colorectal polyposis and hypodontia. Genetic testing confirmed a novel frameshift germline mutation in exon 8 of the *AXIN2* gene. In addition, we provide an updated overview of germline *AXIN2* mutations reported in literature.

**Conclusions:**

Although the number of missing teeth is less severe in our patient than in some previously reported cases, our findings provide additional evidence that missing teeth and gastrointestinal neoplasia are associated with rare pathogenic *AXIN2* germline mutations.

## Background

The canonical WNT signal transduction pathway (also denoted as WNT/β-catenin pathway) is an ancient evolutionary conserved pathway that has a prominent role in embryogenesis and diverse physiological processes, including vascular survival and proliferation, maintenance of the intestinal stem cell niche, odontogenesis, and bone tissue remodelling [1]. In basal conditions, cytosolic β-catenin is continuously subjected to phosphorylation and subsequent proteolytic degradation by action of a multisubunit destruction complex [2]. This destruction complex is composed of the tumour suppressors adenomatosis polyposis coli (APC) and axin inhibition protein 1 (AXIN1) or its homologue AXIN2 (also known as conduction), and the kinases casein kinase 1 (CK1) and glycogen synthase kinase 3β (GSK3β) [2]. Upon stimulation by WNT-ligands, the cell-surface receptors Frizzled and co-receptor LRP5 or LRP6 recruit the cytosolic protein Dishevelled (DVL) that subsequently attracts the multisubunit destruction complex and thereby releases β-catenin [2]. This leads to nuclear translocation of β-catenin where it promotes the transcription of WNT target genes via physical interactions with T cell factor/lymphoid enhancer-binding factor (TCF/LEF) transcription factors [2].

Aberrant WNT signaling, due to germline and somatic mutations in components of this pathway, is a frequent driver in human cancer development and progression [2, 3]. For instance, germline loss-of-function mutations in the *APC* gene underlie familial adenomatous polyposis (FAP) [4, 5], a hereditary condition resulting in numerous colorectal polyps and a very high lifetime risk of colorectal cancer approaching 100% in absence of surveillance and prophylactic proctocolectomy or colectomy. Somatic *APC* mutations are present in approximately 75% of the apparently sporadic colorectal cancers [6].

**Fig. 1:**
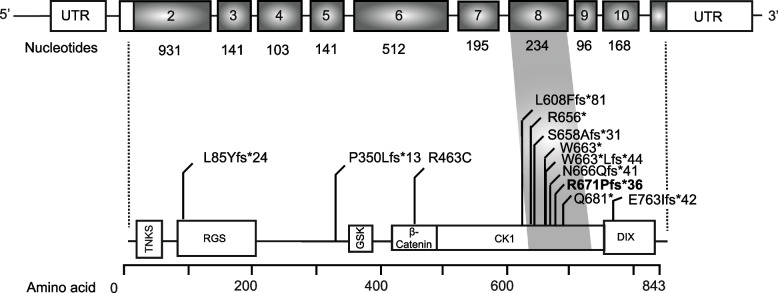
Genomic map of the *AXIN2* gene showing an overview of the germline variants that have been reported so far in literature (NM_004655.3 and NP_004646.3) [7–17, 26]. Numbered boxes represent exons and the in-between lines depict introns. Please note that exon 1 and exon 11 are untranslated

Although rare, loss-of-function germline mutations in the gene *AXIN2* (OMIM 604025; Fig. 1), encoding for a scaffolding component in the β-catenin destruction complex, also predispose to gastrointestinal polyps and cancer [7–17]. So far, approximately 30 patients with germline mutations in the *AXIN2* gene have been described. The limited number of patients makes an accurate genotype-phenotype analysis currently challenging. The clinical phenotype is variable and in addition to gastrointestinal polyps, individuals with *AXIN2* mutations tend to have hypo- and oligodontia [7–17]. Hypodontia, encountered in 2.2% to 10.1% in the general white population, denotes the congenital absence of one to five elements—excluding third molars—in the permanent dentition [18]. Oligodontia, which is very rare with an estimated prevalence of 0.14%, represents a more severe form of dental agenesis with the congenital missing of at least six elements—apart from the third molars—in the permanent dentition [18].

In one previously described family the *AXIN2* mutation not only segregated with colorectal polyposis and oligodontia, the mutation carriers also displayed mild features of ectodermal dysplasia (abnormal development of ectodermally derived structures—skin, sweat glands, hair, and nails) [8]. Furthermore, a single case study more recently suggested that the *AXIN2-*related phenotype spectrum might be expanded by neuroblastomas and gastric adenomas [11]. Somatic *AXIN2* mutations are prevalent in various tissues with carcinoma, including gastro-intestinal [19], skin [20], and adrenal glands.

*AXIN2* itself is a major target gene of WNT signalling. It acts in a negative feedback loop to limit and fine-tune the canonical WNT-signaling [21, 22]. The human *AXIN2* gene is located on chromosome 17q24.1 and encompasses 10 coding exons that generate a protein of 843 amino acids (Fig. 1) [23]. AXIN2 harbours two highly conserved functional domains. The RGS (regulator of G protein signaling; amino acid 81–200) domain in the N-terminus, which mediates physical interaction with APC, and the DIX (Dishevelled/Axin; amino acid 761–843) domain that includes a binding site for DVL. Furthermore, *AXIN2* contains binding motifs for interaction with Tankyrase (amino acid 21–30), GSK3β (amino acid 327–413) and β-catenin (amino acid 413–476).

Here, we report a family with a novel frameshift germline mutation in exon 8 of AXIN2 associated with colorectal polyposis and hypodontia. Furthermore, we provide an updated overview of germline *AXIN2* mutations reported in the literature.

## Case presentation

**Fig. 2:**
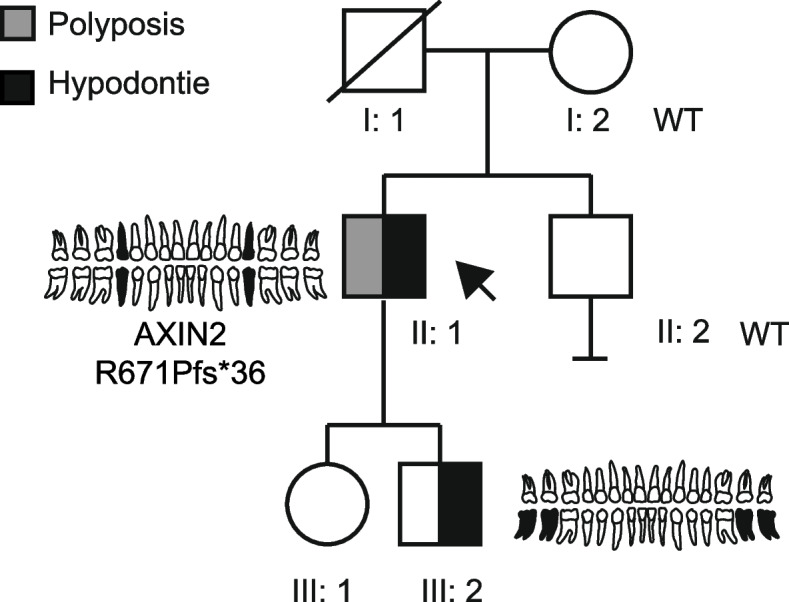
Family pedigree of the proband with hypodontia and polyposis. The proband is indicated by the arrow. Squares and circles represent males and females, respectively. Deceased individuals are indicated by a diagonal line through the symbol. Phenotypes are elaborated by segments showing the presence of hypodontia and polyposis. Schematic odontograms indicate the missing permanent teeth (in black) in the affected individuals

The proband and family members gave consent for the publication of non-identifiable details. The proband (Fig. 2), is a 55-year-old male of mixed European and Chinese descent who was referred by his gastroenterologist to the department of Clinical Genetics in the Amsterdam University Medical Center for genetic evaluation of colonic polyps. A first colono-scopy at the age of 44, was conducted because of changes in the defecation pattern. Two tubular adenomas with low grade dysplasia were identified. The gastroscopy was normal. A subsequent surveillance coloscopy at the age of 54 identified nine tubular adenomas with low grade dysplasia and one sessile serrated lesion without dysplasia. Dental records confirmed that the patient misses four premolars since childhood. The patient has rather scarce body hair which might be due to his partly Asian descent. He had no other possible features of ectodermal dysplasia. The proband has two children. His 15-year-old son is missing two second molars. In addition, agenesis of the third molars seems likely. His daughter has no missing teeth. Given their young age no genetic testing was yet performed in the proband’s children. The proband’s brother has no medical history of gastrointestinal polyps or cancer and has a full dentition. The proband’s father is from Chinese descent. He passed away at the age of 76. He did not have a history of gastrointestinal polyps or cancer. According to the proband, his father required multiple dental prosthesis, which is suggestive of hypo- or oligodontia. No paternale relatives are available for testing of the *AXIN2* gene. The proband’s mother is from European descent. At the age of 90, there is no history of gastrointestinal polyps and she has a full dentition.

### Methodology

A next-generation sequencing (NGS) test has been performed in the proband, utilizing a gene panel covering 23 relevant genes implicated in polyposis and colorectal cancer (*ACVRL1, APC, AXIN2, BMPR1A, ENG, EPCAM, GREM1, MLH1, MLH3, MSH2, MSH3, MSH6, MUTYH, NTHL1, PMS2, POLD1, POLE, PTEN, RNF43, SMAD4, STK11, TSC1,* and *TSC2*). Genomic DNA was extracted from peripheral-blood leukocytes in venous blood samples. DNA libraries for Illumina sequencing were generated using standard protocols from 200 ng to 1 µg of genomic DNA. The libraries were sequenced with paired-end (2 × 150 bp) runs using Illumina MiSeq with a target depth of 30 base coverage. In short, the sequencing reads were aligned to the human reference genome GRCh38 using BWA-MEM (0.7.5). GATK2.8 HaplotypeCaller was used to call SNPs and indels to create gVCF files. Variant Cartagenia Bench Lab V5.0 was used for variant interpretation and prioritization. All exons and 20 flanking intronic nucleotides of the above indicated genes have been analyzed. After gene prioritizing, we were left with one genetic variant in *AXIN2*.

## Results

A novel heterozygous frameshift variant in exon 8 of the *AXIN2* gene was found in the proband (please note that exon 8 was previously also depicted as exon 7 as exon 1 of *AXIN2* is untranslated). The variant, cor-responding to c.2011dup (NM_004655.3), is predicted to cause a frameshift starting at position 671 (denoted as R671Pfs*36 in NP_004646.3) and likely results in a premature termination of translation. No (likely) pathogenic variants have been identified in the other 22 genes tested. This genetic variant was absent in the variation databases Single Nucleotide Polymorphism Database (dbSNP; accessed June 2021) [24] and Genome Aggregation Database (GnomAD) [25]. Furthermore, the variant was not present in an in-house database comprising 1662 individuals who underwent NGS panel testing (January 2015 until December 2022) because of a clinical suspicion of juvenile polyposis (*n* = 14), serrated polyposis (*n* = 22), familial polyposis (*n* = 364), Lynch syndrome (*n* = 518), Cowden syndrome (*n* = 1). The proband’s brother and mother, who are both healthy and have normal dentition, tested negative for the *AXIN2* variant. Relatives of the proband’s father were not available for DNA testing. According on the American College of Medical Genetics and Genomics standards and guidelines for the interpretation of sequence variants, this novel *AXIN2* variant is considered to be pathogenic (class 5).

## Discussion

Here we describe the novel heterozygous pathogenic frameshift variant R671Pfs*36 in exon 8 of the *AXIN2* gene in a 55-year-old man with polyposis and hypodontia. The rather scarce body hair might be due to his partly Asian descent. No other evident features of ectodermal dysplasia were present. Although the number of missing teeth is less severe in our patient than in some previously reported cases, our findings provide additional evidence that missing teeth and gastrointestinal neoplasia are associated with rare pathogenic *AXIN2* germline mutations.

The mutation R671Pfs*36 is predicted to cause a premature termination of protein synthesis and as a consequence this genetic alternation is likely to result in loss of the DIX domain. The DIX domain is not only crucial for interaction with the protein DVL, but is also required for AXIN2 homodimerization. Therefore, in line with previously reported frameshift and truncating mutations affecting exon 8, this novel pathogenic variant will likely impair the inhibitory action of AXIN2 on the WNT sig-naling pathway.

In the *AXIN2* gene exon 8 can be considered as a mutation hotspot, as it is a common location for germline (Fig. 1), as well as somatic mutations (not indicated). The germline mutations mainly concern frameshift mutations, which is not surprising, as exon 8 contains several mononucleotide repeat tracts. In the first reported family, 11 individuals lacking at least 8 permanent teeth and colonoscopic findings ranging from normal to polyposis and colorectal cancer, harboured the nonsense mutation R656* in exon 8 [7]. In the same study, the authors identified another mutation affecting exon 8 (de novo frameshift N666Qfs*41 mutation) in an unrelated 13-year-old patient with severe oligodontia [7]. Whether or not there is a gastrointestinal phenotype in this patient is unknown because of his young age. However, in the meantime this pathogenic variant has been identified in at least four unrelated families in which mutation carriers in addition to hypo- or oligodontia displayed a gastrointestinal phenotype ranging from polyposis to colorectal cancer [12, 16, 17, 26]. Furthermore, the phenotype of oligodontia and colorectal polyposis associated with an exon 8 mutation has been confirmed in three family members harbouring the frameshift S658A*31 mutation [10]. Interestingly, the exon 8 mutation W663*, not only segregated with an autosomal dominant pattern with oligodontia and a variable gastrointestinal neoplastic phenotype, the affected individuals also displayed mild features of ectodermal dysplasia [10]. It remains to be defined whether ectodermal dysplasia is indeed due to the *AXIN2* mutation as this is the only family reported to date with clear features of ectodermal dysplasia.

Although genotype–phenotype correlations are difficult to establish due to the limited number of patients with (likely) pathogenic *AXIN2* mutations, missense mutations that are situated substantially away from exon 8 and affect other protein domains have been described in patients with gastrointestinal polyps and without oligodontia (Table 1). For other cancer predisposition genes, including the *APC* gene, different phenotypes have been described for mutations in different parts of the gene. In light of studying pathogenic *AXIN2* variants and their effect on protein function and clinical phenotype, the currently build *AXIN2* patient registry will be a unique valuable resource [11]. The *AXIN2* patient registry can also contribute to awareness among medical specialists, including dentists that people with oligodontia or families with hypodontia should be referred to a clinical geneticist to discuss *AXIN2* analysis. This is important as people with *AXIN2* mutations can benefit from regular colonoscopy. By removal of polyps the development of colorectal cancer can possibly be prevented. As no comprehensive guidelines are currently available and patients lack evidence of accelerated adenoma-carcinoma progression, we recommend carriers to receive colono-scopy every three years starting from the age of 25 years. The gastroscopy in our patient was negative. In light of the recently reported co-occurrence of germline *AXIN2* mutation with a gastric adenoma, one-off gastroscopy in carriers might gain insight whether gastric adenomas are part of the *AXIN2-*related phenotype spectrum.

**Table 1:** Overview of *AXIN2* mutations in CRC and polyposis with/without hypo- and oligodontia

	**Protein**	**Genomic change**	**Location**	**Individual**	**Sex**	**Age (years)**	**Polyps/CRC (age)**	**Hypo-/oligodontia**	**Number of missing elements**	**Ectodermal dysplasia**	**Reference**
**Missense**	p.R463C	c.1387C > T	Exon 6	II-1	M	51	CRC (51)	-	-	-	[10]
				III-1	M	42	10 polyps, CRC (42)	-	-	-	
				III-2	F	36	30 polyps, CRC (36)	-	-	-	
				III-5	F	43	-	-	-	-	
**Nonsense**	p.R656*	c.1966C > T	Exon 8	II-2	M	54	CRC	+	26	-	[7]
				II-4	M	62	4 polyps	+	29	-	
				II-7	F	57	68 polyps, CRC	+	11	Peg-shaped incisors	
				II-8	M	58	1 polyp	+	29	-	
				III-2	F	35	1 polyp	+	16	-	
				III-4	M	31	3 polyps	+	13	-	
				III-5	F	27	1 polyp	+	21	-	
				III-7	M	35	~ 23 polyps	+	8	Peg-shaped incisors	
				III-8	F	26	-	+	24	-	
				IV-1	M	NR	NR	+	17	-	
				IV-2	F	NR	NR	+	16	-	
	p.W663*	c.1989G > A	Exon 8	II-1	F	68	≥ 5 polyps, CRC (50/59)	+	NR	+	[8]
				II-2	F	65	-	+	NR	+	
				II-3	F	63	-	+	NR	+	
				III-3	F	35	-	+	> 10	+	
	p.Q681*	c.2041C > T	Exon 8		F		> 20 adenomas and CRC (58), ovarian cancer (67)	NR	NR	NR	[13]
	p.P350Lfs*13	c.1049delC	Exon 4	II-1	F	59	CRC	-	-	-	[17]
				II-2	M	48	-	-	-	-	
				III-1	F	55	58 polyps	-	-	-	
				III-2	F	59	5, CRC (74)	-	-	-	
				III-4	F	49	42 polyps	-			
				IV-1	F	NR	Multiple, no details	-	-	-	
				IV-2	F	46	19 polyps	-	-	-	
**Insertion/ deletion**	p.L85Yfs*24	c.254del^a^	Exon 2		F	79	CRC (79)	-	-	-	[14]
	p.L608Ffs*81	c.1822del	Exon 7		F	49	1 stomach polyp, 1 colorectal polyp, 1 neuroblastoma	+	4	-	[11]
	p.S658A*31	c.1972delA	Exon 8	I-1	F	67	> 100 polyps	+	22	-	[9]
				II-3	F	43	5 polyps, CRC	+	NR	-	
				II-4	M	40	> 43 polyps	+	10	-	
	p.W663Lfs*44	c.1987dup	Exon 8		65	NR	CRC (65)	Abnormal dentition	NR	NR	[15]
	p.N666Qfs*41	c.1994dupG^b^	Exon 8		65	M	57 polyps	+	25	-	[12]
					M	13	NA	+	13	-	[7]
				II-2	F	52	CRC (51)	+	NR		[16]
				II-4	F	77	CRC (77)	+	NR		
				III-3	M	58	34 polyps	+	3	Mild ectodermal dysplasia	
				III-5	M	56	CRC (56)	+	5		
				III-9	M	57	NR	+	11	-	
				III-13	M	52	2 polyps	+	5	Brittle hair	
				IV-5	F	26		+	1		
				IV-8	M	27		( +)	1		
				IV-4	M	58	41 polyps	+	6	-	[17]
				IV-8	M	37	5 polyps	+	1	-	
	p.R671Pfs*36	c.2011dupG	Exon 8	II-1	M	44	12 polyps	+	4	-	Current study
**Alternative** **splicing**	r.[= , 2285_2405del] p.(E763Ifs*42)	c.2405G > C	Exon 10		M	60	30 polyps	-	-	-	[17]

GenBank accession no. NM_004655.3 and NP_004646.3

^a^In addition to *AXIN2* c.254del, this patient harboured the synonymous VUS *MSH2* c.1275A > G, (14)

^b^Originally described as c.1995insG in (7)

+ feature present; -, feature absent; NR, feature not reported

Furthermore, with the recent advances of the organoid technology [27], a patient registry also provides an extraordinary opportunity for additional basic and translational studies as patient-derived intestinal organoids – obtained during surveillance colonoscopy – can gain important insight in WNT signaling in a pathophysiological relevant environment. Application of the CRISPR/CAS9 technology in the recently developed dental pulp-organoids [28] provides an alternative to recapitulate the actions of *AXIN2* mutations and study the basics in human tooth organogenesis. In-depth investigation of molecular mechanisms underlying carcinogenesis and tooth development, patient-derived organoids will pave the way for more targeted strategies for personalized cancer medicine and allow for tooth regeneration [29].

## Data Availability

Not applicable.

## Abbreviations

APC: Adenomatosis polyposis coli
AXIN1: Axin inhibition protein 1
AXIN2: Axin inhibition protein 2
CK1: Casein kinase 1
GSK3β: Glycogen synthase kinase 3β
DVL: Dishevelled
TCF/LEF: T cell factor/lymphoid enhancer-binding factor
FAP: Familial adenomatous polyposis
RGS: Regulator of G protein signaling
